# The Suprainguinal Fascia Iliaca Block Prolonging Spinal Anesthesia Duration

**DOI:** 10.7759/cureus.65732

**Published:** 2024-07-30

**Authors:** Jia Yin Lim, Chi Ho Chan

**Affiliations:** 1 Department of Anesthesiology, Sengkang General Hospital, Singapore, SGP

**Keywords:** peripheral nerve block, duration of block, hip surgery, spinal anesthesia, fascia iliaca compartment block (ficb)

## Abstract

Spinal anesthesia is commonly used for lower limb procedures, its duration may be limited with potential complications due to high doses of local anesthetic. This study describes the technique and experience of using suprainguinal fascia iliaca block (SIFI) as an adjunct to spinal anesthesia in an elderly patient undergoing lower extremity surgery. The case presented here involves an 81-year-old female undergoing hip surgery, where a SIFI block was performed prior to the administration of spinal anesthesia. Despite the unexpectedly prolonged surgical duration of approximately 5 hours, the patient remained comfortable, and the surgery was completed without complications.

Subarachnoid block for provision of surgical anesthesia generally lasts between 2 and 3 hours with dose-dependent local anesthetic-related adverse effects. This may hinder the utility of spinal anesthesia in complex cases where extended surgical duration may be expected. The continuous spinal anesthesia and combined spinal-epidural (CSE) are useful techniques to provide consistent peri-operative anesthesia with precise titration of anesthesia levels. However, this presents with a risk of accidental dural puncture with CSE, post-dural puncture headache, and inadvertent drug errors with a spinal or epidural catheter. The judicious use of other adjuvants alongside local anesthetics offers advantages in extending the duration of anesthesia by a modest increment. The integration of spinal anesthesia with SIFI is a promising strategy to extend block duration, reduce peri-operative opioid requirements, and enhance patient outcomes. Overall, SIFI is a safe anesthetic technique for the peri-operative management of hip fracture patients and may present synergistic effects when combined with spinal anesthesia and may prolong the duration of regional anesthesia during unexpectedly prolonged surgery.

## Introduction

The central neuraxial block is often the choice anesthesia for elderly patients undergoing lower extremity surgeries such as total knee replacements and surgical fixation of hip fractures. However, the utility of this technique is restricted by the complexity of surgery and duration of surgery. Subarachnoid block for provision of surgical anesthesia generally lasts between 2 and 3 hours, and the optimal dose of local anesthetic affecting its duration of action has a narrow reliability margin to yield the desired effect [1]. On the contrary, high doses of local anesthetic or additives in the intrathecal space can lead to undesirable complications such as cardiovascular instability, total spinal and potentially increased risk of neurotoxicity, or transient neurological symptoms [2,3].

Fascia iliaca compartmental block is a regional anesthesia technique used for the provision of pain relief to the lower extremities and pelvis. This anterior lumbar plexus block aimed to anesthetize the femoral nerve, the lateral femoral cutaneous nerve, and the obturator nerve which provides analgesia to the anterior hip capsule, the femoral shaft, and surgical skin incisions. While the effectiveness of interventions in reducing peri-operative pain, minimizing opioid consumption, improving early mobilization, and shortening hospital stays after hip surgery is well established, evidence regarding their impact on prolonging the duration of spinal anesthesia remains limited [4,5]. This study presents a case of suprainguinal fascia iliaca (SIFI) block prolonging the duration of spinal anesthesia.

## Case presentation

An 81-year-old Chinese female, 152 cm tall, weighing 55 kg, with the background of previous closed reduction and surgical fixation of left subtrochanteric hip fracture with the TFN-ADVANCED Proximal Femoral Nailing System (TFNA) (Raynham, MA: DePuy Synthes) one year ago, was admitted after she slipped and fell over her wet toilet floor and suffered a left femoral shaft periprosthetic fracture. Her medical history included hypertension, hyperlipidemia, type 2 diabetes mellitus, stage 3A chronic kidney disease, stable coronary artery disease on aspirin and clopidogrel, and sinus node dysfunction with pacemaker insertion 11 years ago. Prior to admission, she was able to perform activities of daily living independently and ambulate around the house. The patient was planned for a left TFNA removal and insertion of an RFN-ADVANCED Retrograde Femoral Nailing System (RFN) (Raynham, MA: DePuy Synthes) seven days after suspension of anti-platelet therapies in view of potential intra-operative significant blood loss. The expected duration of the surgery was estimated to be about 150 minutes. A pacemaker check was performed prior to surgery, and a magnet was available to convert the pacemaker to asynchronous mode if required intra-operatively.

On the day of surgery, the patient was placed on standard monitoring with non-invasive blood pressure, electrocardiogram, and pulse oximetry. An ultrasound-guided left suprainguinal fascia iliaca compartmental block was performed with 25 mL of 0.4% ropivacaine prior to spinal anesthesia. Spinal anesthesia was performed at L3-L4 intervertebral space in the right lateral position using 0.5% plain bupivacaine 2.3 mL and fentanyl 15 mcg. A sensory block level of T8 was achieved. The patient was turned back to the supine position 5 minutes after intrathecal injection for surgical positioning and preparation. Propofol target-controlled infusion with the Schneider model was used to maintain light sedation for patient comfort, and the patient remained comfortable at the maintained effect-site target of 0.5 mcg/mL.

Surgery was complicated by difficulty in removal of the previous TFNA and nail and limited range of movement after insertion of the RFN requiring several adjustments of the implant, resulting in unexpectedly longer surgical time. The total time taken from the time of spinal anesthesia to the last stitch was 4 hours and 55 minutes. Despite the prolonged surgical time, the patient remained comfortable with minimal sedation and the surgery was completed uneventfully.

Post-operatively, the patient remained well and participated in physiotherapy from post-operative day one, making gradual improvements over her hospital stay. She was discharged well to the community hospital one week after surgery for further rehabilitation.

Block technique description

**Figure 1:**
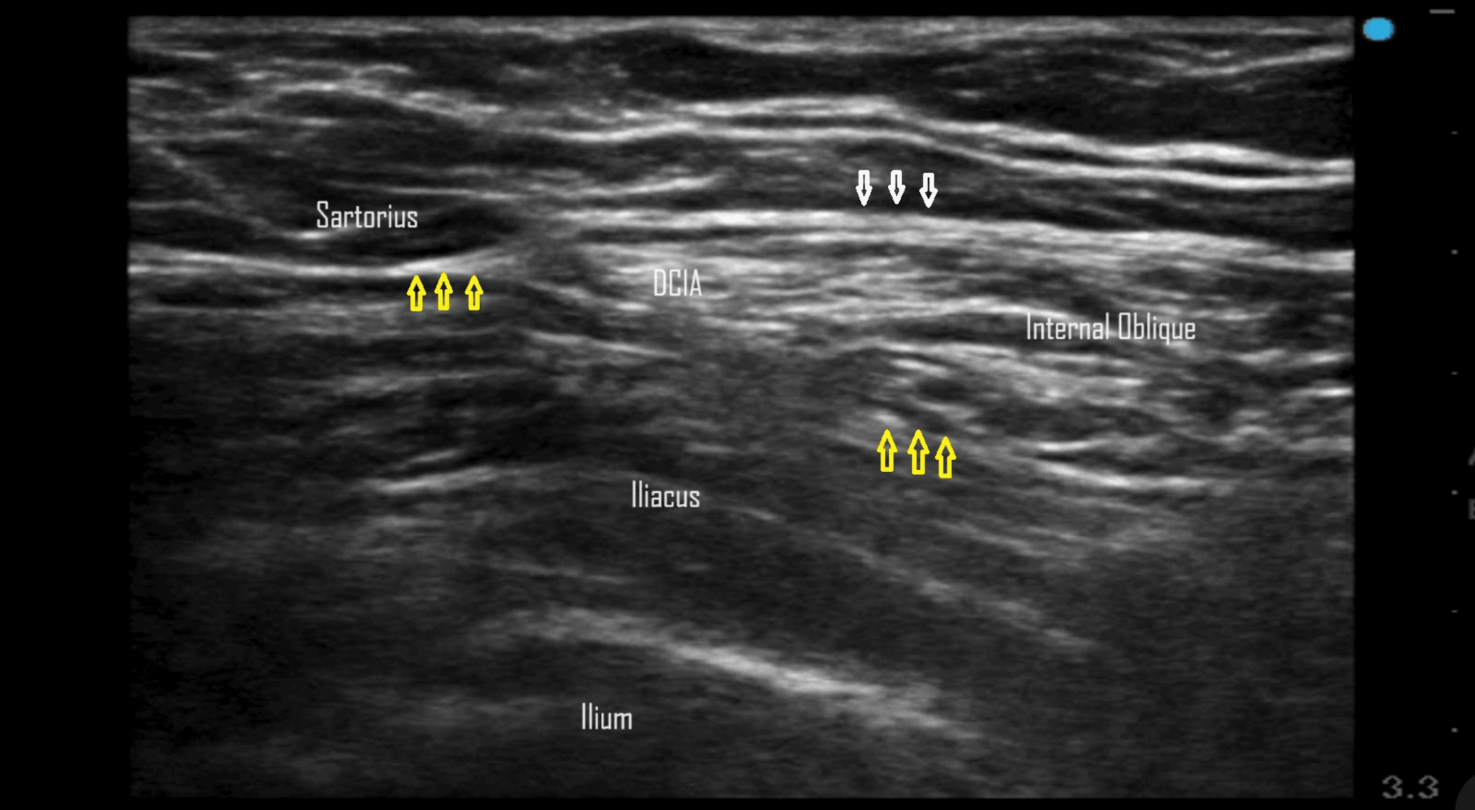
Suprainguinal fascia iliaca compartmental block. Fascia lata (white arrows), fascia iliaca (yellow arrows), and deep circumflex iliac artery (DCIA).

**Figure 2:**
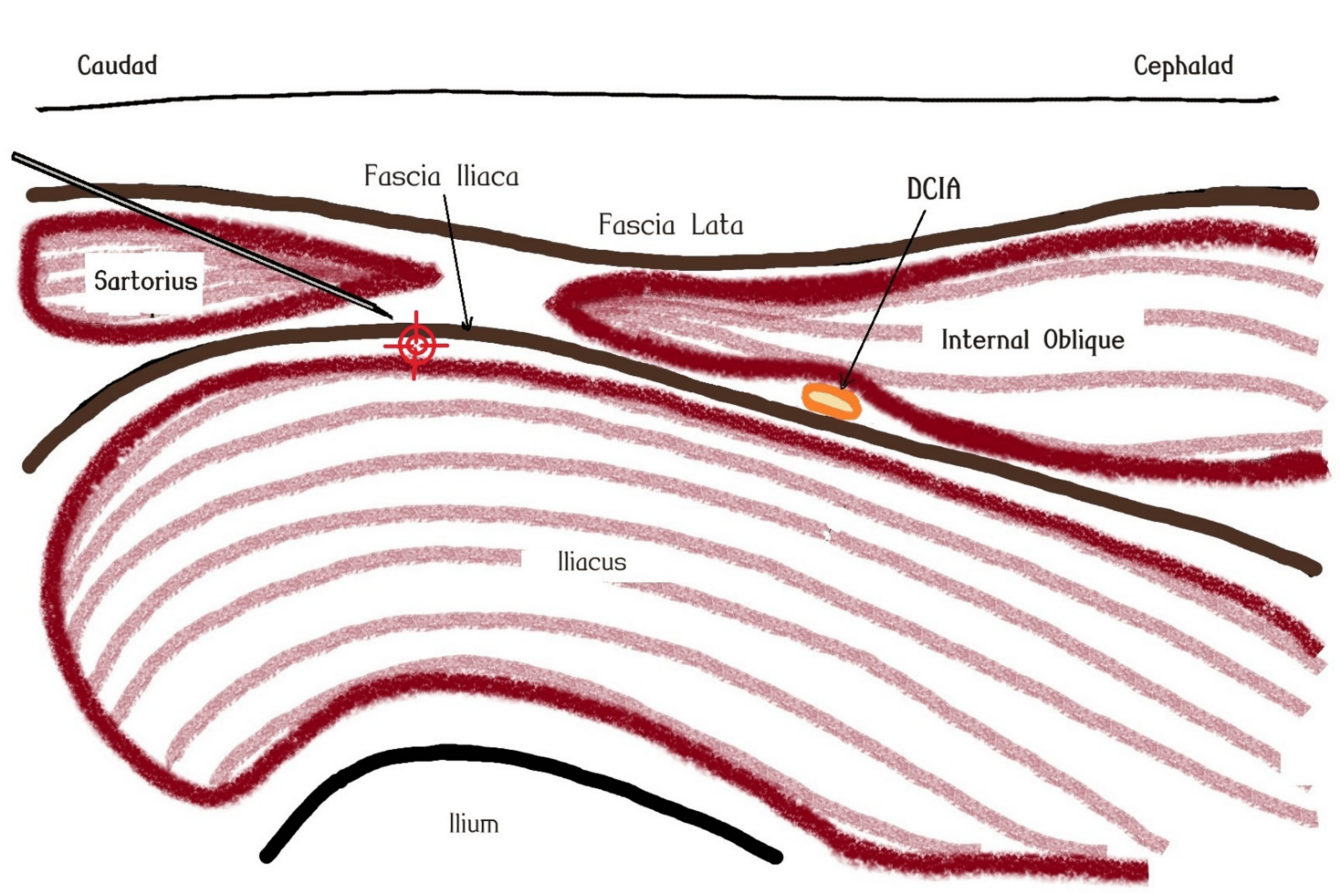
Schematic diagram of suprainguinal fascia iliaca compartmental block. Deep circumflex iliac artery (DCIA), plane of expected local anesthetic disposition (red target sign). The image is created by the authors of this study.

In the supine position, a 12-5  MHz linear ultrasound probe was placed over the inguinal ligament on the inguinal crease to identify the femoral artery, fascia lata, fascia iliaca covering the iliacus muscle, and femoral nerve. The ultrasound probe was slid laterally and the most superficial part of the iliacus muscle is placed in the center of the ultrasound image. The ultrasound probe was then rotated clockwise, with the medial side of the ultrasound probe pointing towards the umbilicus. The “bow-tie sign” consisting of the iliacus muscle, sartorius muscle, and internal oblique muscle was identified (Figures 1, 2). Under sterile technique, block site was cleaned with 2% chlorhexidine solution. Using the ultrasound-guided in-plane technique, the local anesthetic was deposited under the fascia iliaca. A 23 gauge B Braun Sterican (Melsungen, Germany) hypodermic needle was used to deposit local anesthetic subcutaneously to provide local anesthesia for patient comfort. A 100 mm PAJUNK SonoPlex STIM (Geisingen, Germany: PAJUNK) insulated needle was used to perform the block. The fascia iliaca compartment was then hydrodissected with local anesthetic and the needle was advanced proximally, moving into the hydrodissected fluid spaces, until the entire needle was inserted into the skin (Video 1). A total of 25 mL of 0.4% ropivacaine was administered.

**Video 1:** Fascia iliaca compartmental block.

## Discussion

The hip joint receives sensory innervation from the femoral nerve (anterior hip joint), obturator nerve (medial hip joint), and articular branches of the sciatic nerves (posterior hip joint) and regional anesthesia techniques covering these nerves can provide analgesia for patients with hip fracture. Moreover, the lateral cutaneous branch of the subcostal nerve provides sensory innervation of the skin over the lateral thigh and may be useful to provide analgesia for the incision site of hip fracture surgery [6]. Over the last decades, there has been a significant emphasis on and evolution in anterior approaches to lumbar plexus regional anesthesia for hip fracture and hip fracture surgery. This shift is due to the posterior lumbar plexus blocks, such as the psoas compartment block, being considered advanced techniques with considerable risk of complications. The “3-in-1” block was described in 1973, performed at the level of the inguinal ligament to provide anesthesia to the femoral, obturator, and lateral femoral cutaneous nerves [7]. This technique was later refined with the introduction of ultrasound guidance. Decades later, the suprainguinal approach targeting the anterior lumbar plexus at the level of the psoas muscles was introduced and has since been adapted and advocated by many specialists [8].

Subarachnoid block for provision of surgical anesthesia generally lasts between 2 and 3 hours, depending on the local anesthetic dose administered. However, dose-dependent local anesthetic-related adverse effects such as hypotension, urinary retention, and prolonged motor blockade affecting post-operative mobilization may be observed [9]. This may hinder the utility of spinal anesthesia in complex cases where extended surgical duration may be expected. In such cases, continuous spinal anesthesia and combined spinal-epidural (CSE) may be useful techniques where prolonged surgical duration is expected. They provide consistent peri-operative anesthesia, reducing the need for repeated injections and conversion to general anesthesia. It also allows for precise titration of anesthesia levels for better peri-operative hemodynamic management. However, potential drawbacks of such a technique include the risk of accidental dural puncture with CSE, post-dural puncture headache, and inadvertent drug errors with a spinal or epidural catheter.

Providentially, in addition to the use of local anesthetic, adjuvants can be used to enhance the effects of spinal anesthesia and prolong the duration of spinal anesthesia. Opioids, such as fentanyl and morphine, are frequently employed due to their potent analgesic properties and ability to synergistically inhibit pain transmission. While intrathecal opioid administration offers significant benefits in reducing the required dose of local anesthetic and improving post-operative analgesia, it also poses risks, including pruritis and respiratory depression [10]. Clonidine, an alpha-2 adrenergic agonist, enhances spinal anesthesia by blocking pain transmission via the C and A delta fibers. However, at standard doses of 1-2 mcg/kg, it may cause bradycardia and sedation. Dexamethasone, a synthetic glucocorticoid, when used as a perineural adjuvant, can increase sensory block duration following spinal anesthesia by approximately 30 minutes [11]. Several other adjuvant therapies have been described including the use of ketamine, midazolam, and non-steroidal anti-inflammatory drugs (NSAIDs). The judicious use of these adjuvants alongside local anesthetics offers advantages in extending the duration of anesthesia by a modest increment. Nonetheless, it represents a valuable strategy for enhancing anesthesia effectiveness and improving patient outcomes.

The fascia iliaca compartment is a potential space between fascia iliaca anteriorly and iliopsoas muscle posteriorly where the lumbar plexus passes through. Local anesthetic injected within this compartment therefore provides anesthesia of the femoral, obturator, and lateral cutaneous nerves from the lumbar plexus. In addition to the pre-operative and post-operative benefits of fascia iliaca blocks for hip fracture patients, fascia iliaca compartmental blocks have been shown to be useful during intra-operative hip fracture surgery. In conjunction with general anesthesia, it could significantly reduce intra-operative opioid requirements. Several reports have even demonstrated the feasibility of employing fascia iliaca compartment block (FICB) as the primary intra-operative anesthesia for hip fracture surgeries, complemented with mild-to-moderate sedation [12,13]. The integration of spinal anesthesia with SIFI emerges as a promising strategy, offering the potential to extend block duration and improve peri-operative analgesia. This approach not only streamlines the procedural aspects, facilitates comfortable positioning for the administration of spinal anesthesia, and improves peri-operative patient experience, but it also holds promise in mitigating complications related to sedation and peri-operative opioid use in hip fracture patients, a population susceptible to delirium. As illustrated in our case, the addition of suprainguinal fascia iliaca compartmental block may be able to significantly prolong the duration of regional anesthesia and avoid the need for additional analgesic and sedation or conversion to general anesthesia in the event of unanticipated prolonged surgical duration.

## Conclusions

Suprainguinal fascia iliaca (SIFI) block allows for continuous pain management and smoother surgical experience for the peri-operative mangement of hip fracture patients. As presented in our case, SIFI block may also present synergistic effect when combined with neuraxial anesthesia. This may prolong the duration of regional anesthesia during unexpectedly prolonged surgery avoiding the need for additional analgesia or conversion to general anesthesia. This combined approach may enhance patient comfort and outcomes, particularly in the vulnerable hip fracture population.
